# Systemic Embolism and Clinically Significant Bleeding Events in Older Adults with Nonvalvular Atrial Fibrillation After Treatment with Direct Oral Anticoagulants and Warfarin: A Retrospective Cohort Study in Japan

**DOI:** 10.3390/pharmaceutics16121515

**Published:** 2024-11-25

**Authors:** Daichi Yaguchi, Shoji Sera, Akira Okada, Yuki Nishimura, Satoshi Tamaru, Naomi Nagai

**Affiliations:** 1 Laboratory of Regulatory Science, Faculty of Pharmacy, Musashino University, Nishi-tokyo, Tokyo 202-8585, Japan; dyaguchi@alnylam.con (D.Y.);; 2 Medical Affairs Department, Alnylam Japan KK, Chiyoda-ku, Tokyo 100-6211, Japan; 3 Research Institute of Pharmaceutical Sciences, Musashino University, Nishi-tokyo, Tokyo 202-8585, Japan; 4 Clinical Research Support Center, Mie University Hospital, Tsu 514-8507, Mie, Japan

**Keywords:** direct oral anticoagulant agents, nonvalvular atrial fibrillation, elderly patients, Japanese, database research, bleeding

## Abstract

**Background/Objectives:** Anticoagulant therapy, particularly the use of direct oral anticoagulant agents (DOACs), is recommended for patients with nonvalvular atrial fibrillation (NVAF). This multicenter observational retrospective cohort study aimed to assess the efficacy and safety of DOACs compared to warfarin in Japanese patients aged 75 years and older with NVAF. **Methods:** Data from the Mie-Life Innovation Promotion Center Database were used to collect medical information on the patients. The cumulative incidences of clinically significant bleeding events and systemic embolic events (SEEs) were analyzed. **Results:** This study included 1787 older adult patients, of whom 1321 received DOACs (edoxaban: 428; apixaban: 586; dabigatran: 105; rivaroxaban: 202) and 466 receiving warfarin. There were no statistically significant differences in the cumulative incidence of bleeding events between the DOAC- and warfarin-treated groups. However, a statistically significant difference was observed for SEEs, with dabigatran showing a significantly lower incidence compared to warfarin. **Conclusions:** The incidence rates of bleeding events for individual DOACs were comparable to those for warfarin. Additionally, a history of vascular disorders was identified as a risk factor for bleeding events in the DOAC-treated group (hazard ratio (HR): 1.83, 95% confidence interval (CI): 1.16–2.88, *p* < 0.01) and warfarin-treated group (HR: 1.80, 95% CI: 1.15–2.84, *p* < 0.01). Based on real-world data, the overall efficacy and safety of DOACs were generally comparable to warfarin.

## 1. Introduction

In recent years, the prevalence of atrial fibrillation (AF) in Japan has been rising due to the aging population. It is estimated that by 2050, approximately 1.03 million people will have AF, accounting for 1.1% of the total population [1]. While valvular AF, primarily caused by rheumatic valvular disease, was once common, nonvalvular AF (NVAF), driven mainly by lifestyle-related conditions such as hypertension and diabetes, has become the predominant form [2]. Before the widespread adoption of anticoagulation therapy for stroke prevention in NVAF patients, individuals with NVAF had a fivefold higher risk of stroke compared to those in normal sinus rhythm, and approximately one-third of NVAF patients experienced a stroke [3,4]. Moreover, low-intensity anticoagulation with warfarin reduced the risk of cerebral infarction in NVAF patients without increasing the risk of major hemorrhage [5].

Among direct oral anticoagulant agents (DOACs), edoxaban tosylate hydrate (edoxaban), apixaban, dabigatran etexilate methanesulfonate (dabigatran), and rivaroxaban have been shown to significantly lower the risk of intracranial bleeding in NVAF patients compared to warfarin in multiregional clinical trials. Additionally, rivaroxaban exhibited comparable efficacy to warfarin in phase III trials conducted in Japan [6,7,8,9,10]. As a result, DOACs, alongside warfarin, play an important role in modern anticoagulant therapy [1]. However, DOACs are contraindicated in patients with a creatinine clearance below 15 mL/min and those on maintenance dialysis, where warfarin remains the preferred option [7].

Multiregional clinical trials have reported a higher risk of bleeding with DOACs in elderly patients and Asians compared to non-Asians [11,12,13,14]. This raises concerns about an increased risk of bleeding in Japanese patients, who tend to be older and generally of lower body weight [15,16]. Notably, elderly patients are often excluded from clinical trials, including those for DOACs, leaving gaps in understanding the impact of aging and low body weight on the efficacy and safety of these agents. The lack of robust data on elderly Japanese patients highlights the importance of postmarketing surveillance and the need for further evidence from clinical practice in Japan [17,18,19,20].

This retrospective cohort study used an electronic medical record database, focusing on Japanese patients with NVAF aged 75 years or older who presented characteristics that may increase their risk of bleeding, such as advanced age, low body weight, and impaired renal function. These patient groups had not been sufficiently studied in clinical trials during the development of DOACs. This study aimed to compare the cumulative incidences of bleeding and systemic embolism events (SEEs) between patients treated with DOACs, following the approved dosage regimen and adjustments in Japan, and those treated with warfarin. Additionally, this study aimed to identify risk factors associated with bleeding events, which are a significant concern when administering DOACs to elderly patients, using data from real-world clinical practice in Japan.

This study provides additional real-world evidence on the efficacy and safety of DOAC therapy in elderly Japanese NVAF patients, particularly those with lower body weight and reduced renal function, which may not have been thoroughly investigated in clinical trials. In Japan, individuals aged 75 and older are classified as “late-stage elderly”. Current arrhythmia treatment guidelines [1] identify ages 75 and older as a risk factor for stroke, underscoring the relevance of focusing on this population.

## 2. Materials and Methods

### 2.1. Database

The study used the Mie-Life Innovation Promotion Center Database (Mie-LIP DB), an integrated medical information system designed for the Mie region. This electronic database contains medical information from electronic medical records managed by Mie University and stores data from multiple participating medical institutions. Data are stored separately for each hospital on a server, with a focus on patient care achieved through collaboration between Mie Prefecture, Mie University Hospital, and nine core regional hospitals: Kuwana City Medical Center, Mie Prefectural General Medical Center, Suzuka Kaisei Hospital, Suzuka General Hospital, Mie University Hospital, Saiseikai Matsusaka General Hospital, Japanese Red Cross Ise Hospital, Owase General Hospital, and Kinan Hospital [21].

To ensure privacy, all data were anonymized by removing personal identifiers before being compiled and analyzed to produce results across various disease areas. The primary objective of this database is to improve the quality of medical care and contribute to the advancement of healthcare practices through valuable data analysis [22]. The authors accessed the anonymized data for research purposes on 9 December 2021, and had no access to information that could identify individual participants during or after data collection.

### 2.2. Study Population

In this study, patients diagnosed with NVAF were selected if their medical information was available for at least 180 days from the start of the follow-up, defined as the date of their first hospitalization or outpatient record. The medical records were selected from a cohort of patients with inpatient and outpatient prescription data covering the period from 1 January 2016 to 31 December 2018. Patients with a history of SEEs or hemorrhagic events within 30 days prior to the follow-up start date, or whose follow-up periods began and ended on the same day, were excluded from the analysis.

The study specifically focused on elderly patients aged 75 years or older who were treated with the approved dosage of DOACs in Japan and compared them to those treated with warfarin. In Japan, individuals aged 75 years or older are categorized as “late elderly”, and this age group is considered a risk factor in the CHADS score, which is used to evaluate the risk of stroke [1,23]. The inclusion criteria for this study were patients aged 75 years or older, those receiving either DOACs or warfarin, and patients using DOACs at the approved “usual dose” or “dose reduction criterion” (Table 1).

### 2.3. Patients’ Information

The baseline characteristics of the patients were collected, including age, gender, concomitant medications (such as aspirin, adenosine diphosphate receptor [ADPR] inhibitors, and P-glycoprotein inhibitors), and medical history (including congestive heart failure/left ventricular systolic dysfunction, hypertension, diabetes, stroke, vascular disorders such as myocardial infarction, peripheral arterial disease, and aortic plaque, as well as gastrointestinal bleeding). Additionally, renal function was assessed using creatinine clearance (Ccr), and the dose of DOAC was categorized as either standard treatment or reduced-dose treatment.

### 2.4. Outcomes

The primary outcomes of the study were the cumulative incidence of bleeding events and SEEs. These outcomes were calculated for each treatment agent in the DOAC-treated group and warfarin-treated group. Bleeding events were defined as instances requiring transfusion, as well as intracranial bleeding, intraocular bleeding, upper gastrointestinal bleeding, and lower gastrointestinal bleeding. Although hemoglobin (Hb) or hematocrit (Hct) data were not collected, we categorized bleeding events not strictly classified as major bleeding as clinically significant bleeding events. A detailed definition of these events is provided in Table S1. Efficacy events were defined as systemic embolism.

### 2.5. Statistical Analysis

For demographic data, frequencies and proportions were calculated for discrete data in each group, while descriptive statistics, including mean and standard deviation, were used for continuous data. The time from the start of the observation to the occurrence of bleeding and SEEs or censoring was recorded, and the cumulative incidence up to 180 days, during which many patients were traceable, was compared with that of the warfarin-treated group. Additionally, secondary endpoints included hazard ratios (HRs) to assess the risk factors for bleeding events associated with each DOAC and warfarin. To analyze bleeding events and SEEs, the cumulative incidence for each drug was calculated using the Kaplan–Meier method and compared between the DOAC- and warfarin-treated groups using the log-rank test. A Cox proportional hazards model was constructed to assess the risk factors associated with bleeding events. The following factors were included as explanatory variables: concomitant use of ADPR inhibitors, concomitant use of P-glycoprotein inhibitors, concomitant use of aspirin, age, gender, weight, Ccr, and medical history (congestive heart failure/left ventricular systolic dysfunction, hypertension, diabetes, stroke, vascular disorders, and gastrointestinal bleeding).

HRs for bleeding events were calculated for each explanatory factor, and the factors that remained in the final model were identified as risk factors using a stepwise method based on *p*-values. A *p*-value of less than 0.05 was considered statistically significant. Statistical analyses were performed using the R Commander plug-in for the EZR package (version 4.0.2, RcmdrPlugin.EZR) [28].

### 2.6. Ethical Considerations

This study was approved by the Research Ethics Committee of Musashino University and the Clinical Research Ethics Review Committee of Mie University Hospital (approval numbers R3-05, R4-7, R5-4, and H2021-225). According to the ethical guidelines for medical and biological research involving human subjects set by the Ministry of Education, Culture, Sports, Science, and Technology, the Japanese Ministry of Health, Labor and Welfare, and the Ministry of Economy, Trade and Industry, written informed consent was not obtained from each patient for the use of their clinical records in this study.

To ensure transparency, we published all relevant information regarding this study and provided each patient the opportunity to decline participation by posting the details on the institutions’ homepages. Given the lack of consent, patient records and information were anonymized and treated with strict confidentiality to maintain privacy. This study was conducted in accordance with the ethical principles for medical research outlined in the Declaration of Helsinki (1964) and its subsequent revisions. The study did not include minors.

## 3. Results

### 3.1. Patients’ Characteristics

This study included 1787 patients with NVAF who were aged 75 years or older and treated with DOACs or warfarin. Among them, 428 patients were treated with edoxaban, 586 with apixaban, 105 with dabigatran, 202 with rivaroxaban, and 466 with warfarin. Additionally, 466 patients were treated with warfarin. The demographic characteristics of patients in the warfarin-treated group and each DOAC-treated group were examined. 

The results revealed that the mean age was 82 years in the edoxaban-treated group, 83 years in the apixaban-treated group, 81 years in the dabigatran-treated group, 81 years in the rivaroxaban-treated group, and 83 years in the warfarin-treated group. The mean weight was 50.00 kg in the edoxaban-treated group, 49.70 kg in the apixaban-treated group, 53.40 kg in the dabigatran-treated group, 51.80 kg in the rivaroxaban-treated group, and 54.40 kg in the warfarin-treated group. Furthermore, the mean Ccr level was 43.67 mL/min in the edoxaban-treated group, 40.83 mL/min in the apixaban-treated group, 50.11 mL/min in the dabigatran-treated group, 42.55 mL/min in the rivaroxaban-treated group, and 43.09 mL/min in the warfarin-treated group (Table 2).

### 3.2. Bleeding Events

**Figure 1:**
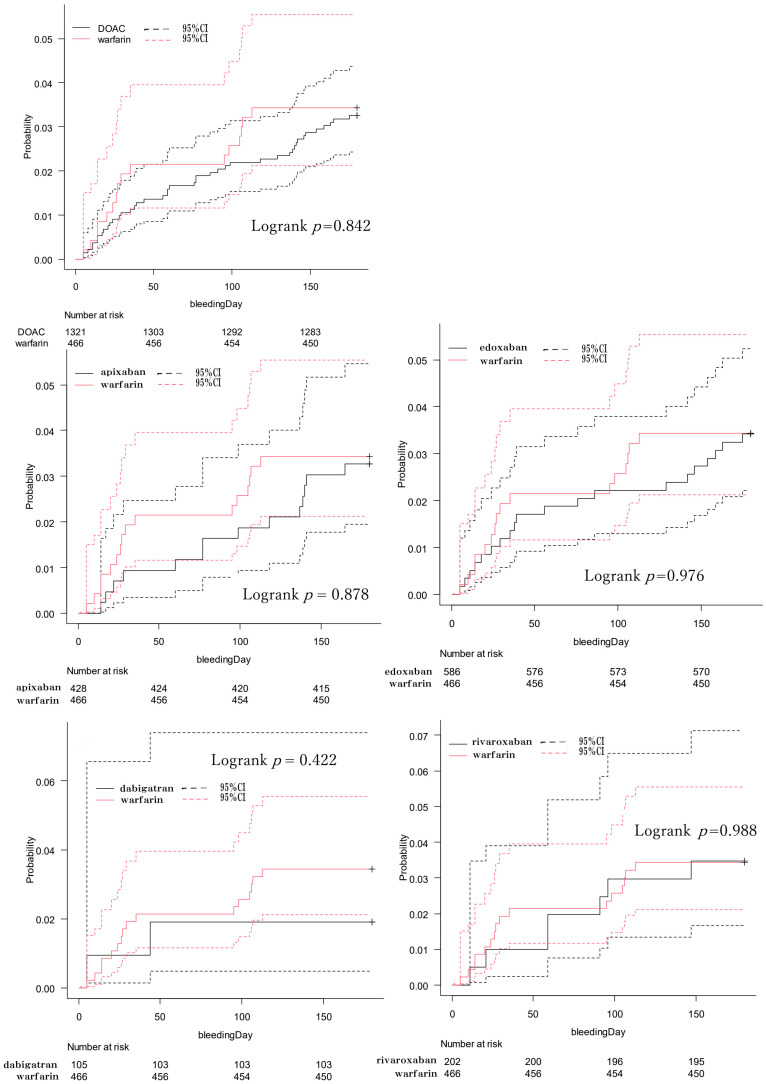
Kaplan–Meier curves for the cumulative incidence up to 180 days of bleeding events.

The Kaplan–Meier curves for the cumulative incidence of bleeding events for each DOAC are depicted in Figure 1. The analysis revealed no significant differences in the cumulative incidence of bleeding events between the DOAC-treated and warfarin-treated groups, as well as among the individual DOAC groups compared to warfarin. The incidence rate of bleeding events up to 180 days was 3.40% (95% confidence interval (CI): 2.10–5.50) in the warfarin-treated group and 3.30% (95% CI: 2.40–4.40) in the DOAC-treated group. Follow-up was successful for many patients up to the 6-month mark; however, more than half of the patients were lost to follow-up by the 1-year mark. For reference, the Kaplan–Meier curve extending up to 1000 days is also shown in Figure S1. Additionally, Table S2 provides a breakdown of the bleeding events observed in each treatment group. Comparisons of the bleeding event rates at 180 days between the individual DOAC and warfarin groups yielded similar results (Table 3).

### 3.3. Systemic Embolism Events (SEEs)

**Figure 2:**
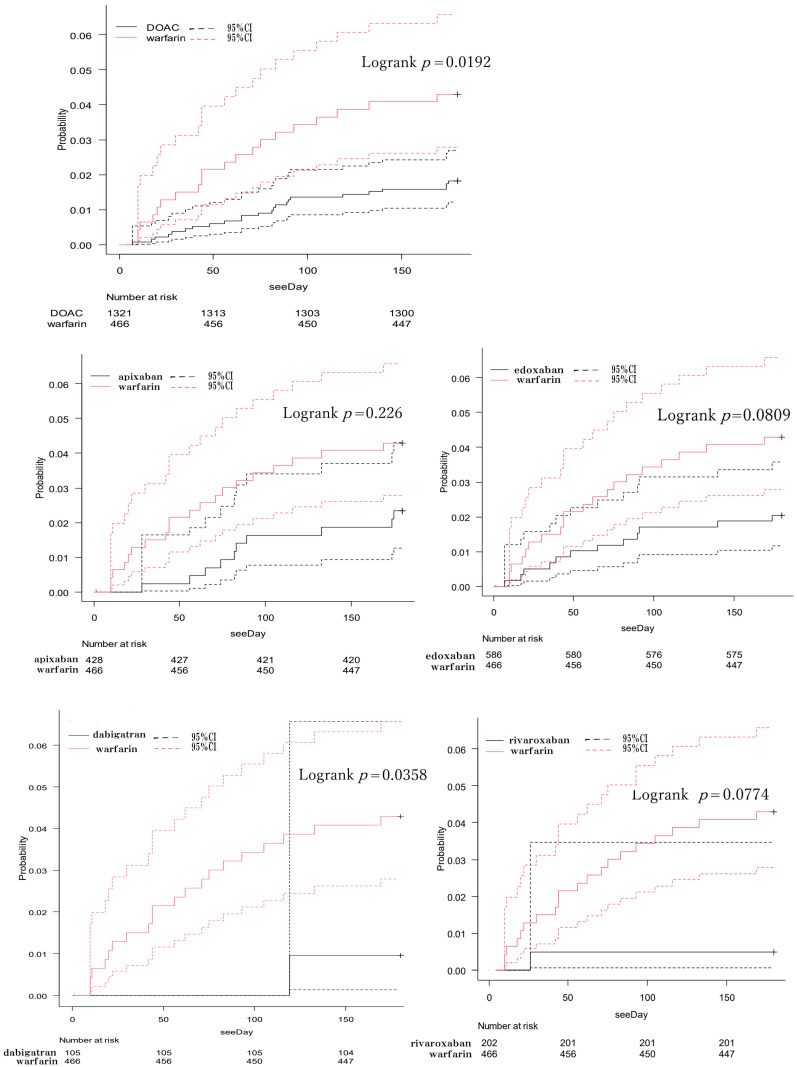
Kaplan–Meier curves for the cumulative incidence up to 180 days of systemic embolism events.

The cumulative incidence of SEEs is depicted in Figure 2. The analysis revealed a statistically significant difference in the cumulative incidence of SEE events up to 180 days between the DOAC and warfarin treatment groups (*p* = 0.0192). When comparing individual DOACs with warfarin, only dabigatran demonstrated a statistically significant difference (*p* = 0.0358). The event rate at 180 days was 4.30% (95% CI: 2.80–6.60) in the warfarin-treated group and 1.80% (95% CI: 1.20–2.70) in the DOAC-treated group (Table 4). For reference, the Kaplan–Meier curve up to 1000 days is also shown in Figure S2. The statistically significant difference in the cumulative incidence of SEE events between the DOAC and warfarin treatment groups observed up to 180 days was not maintained when calculated up to 1000 days. Additionally, Table S3 provides a breakdown of the SEE events observed in each treatment group.

### 3.4. Risk Factors for Bleeding Events

Table 5 summarizes the analysis of risk factors for bleeding events in the DOAC-treated group. A history of vascular disorders was identified as a significant risk factor (HR: 1.83, 95% CI: 1.16–2.88, *p* < 0.01). For individual DOACs, Ccr levels in the edoxaban group (HR: 0.96, 95% CI: 0.93–1.00, *p* = 0.03), and a history of stroke/transient ischemic attacks (HR: 2.75, 95% CI: 1.12–6.75, *p* = 0.03) and vascular disorders (HR: 5.18, 95% CI: 1.67–16.09, *p* < 0.01) in the rivaroxaban group were significant risk factors for bleeding events. However, no risk factors were observed in the apixaban- or the dabigatran-treated groups. Additionally, in the warfarin group, vascular disorders were identified as a significant risk factor for bleeding disorders (HR: 1.80, 95% CI: 1.15–2.84, *p* < 0.01).

## 4. Discussion

The final reports of postmarketing surveillance studies on edoxaban and rivaroxaban indicated that patient characteristics, including age, weight, and renal function, were similar between the Mie region and nationwide observations in Japan [29,30]. Additionally, a study by Tanizawa et al., using the Mie-LIP database, further supports that NVAF patients in the Mie region exhibit characteristics comparable to those observed across Japan [21].

In contrast to clinical trials that included younger and heavier patients with better renal function, postmarketing surveillance and the study by Tanizawa et al. suggest that real-world clinical practice in Japan involves a larger proportion of NVAF patients receiving DOACs who are older, have lower body weight, and exhibit reduced renal function. This discrepancy indicates that the efficacy and safety of DOACs in these populations may not have been fully examined during drug development, highlighting the necessity of accumulating evidence from Japanese medical practice, particularly concerning older adult patients.

The study population, extracted from the Mie-LIP database, comprised patients aged 75 and older, with an average age in their 80s, an average weight of 50 kg, and Ccr levels around 45 mL/min, putting them at higher risk of bleeding than those in postmarketing surveillance. Since almost half of the patients in the edoxaban and rivaroxaban surveillance studies were also 75 years or older, a significant number of patients with similar characteristics exist in actual clinical practice in Japan.

In this study, the population, the occurrence of bleeding events, and SEEs were analyzed when DOACs were administered according to the approved dosage and dose adjustments. The cumulative incidence of bleeding events with DOACs followed a similar trend to that observed with warfarin, while a statistically significant difference was observed for SEEs. When comparing SEEs between individual DOACs and warfarin, only dabigatran showed statistically significant positive results compared to warfarin. The 180-day event incidence rate of SEEs for dabigatran was low at 0.1% (95% CI: 0.1–6.6), likely due to the small number of patients in the dabigatran-treated group and the dose reduction criteria for dabigatran in patients aged 70 or older. In this study, 97.1% of the patients received the reduced dose, contributing to the lower 180-day event incidence rate.

These findings suggest that DOACs are as effective and safe as warfarin in real-world settings for elderly Japanese patients who can receive DOACs at the approved dose and dosage regimen. The yearly incidence of SEEs with apixaban and rivaroxaban was about double the incidence reported in a large American cohort, where more than 60% of participants were over the age of 75, and higher than in American Medicare beneficiaries with a median age of 77 [31,32]. This may be attributed to the inclusion of only patients aged 75 and older in our study, as well as the fact that many patients were underweight and had low renal function. While patient weight and renal function were not specified in the aforementioned US study, only around 25% of patients were on reduced doses. In contrast, 66.9% of apixaban patients and 58.4% of rivaroxaban patients were on reduced doses in this study. This difference in the proportion of patients on reduced doses may reflect variations in renal function and weight between the patients in these studies.

In AF patients, assessing bleeding risk is crucial to prevent complications from anticoagulant therapy. The HAS-BLED score, outlined in the 2020 Arrhythmia Pharmacotherapy Guidelines, evaluates bleeding risk based on factors such as hypertension (systolic blood pressure exceeding 160 mmHg), renal and hepatic dysfunction, stroke, bleeding history, an unstable international normalized ratio, age over 65, certain medications (antiplatelets, anti-inflammatories, analgesics), and alcohol consumption [1]. The findings in our study align with the HAS-BLED score, as reduced Ccr levels and a history of cerebral infarction were identified as risk factors for bleeding in the DOAC-treated group. This is consistent with the finding that renal function is adopted as a dose reduction criterion for all DOACs [24,25,26,27].

In our study, patients with a history of vascular disorders, such as myocardial infarction, peripheral arterial disease, or aortic plaque, displayed a higher risk of bleeding. The presence of preexisting vascular disorders is not specifically mentioned in the current instructions for use in package inserts or clinical guidelines. Since many elderly patients have a history of vascular disorders, it is essential to consider their medical history. Notably, impaired endothelial function, commonly observed in patients with preexisting vascular disorders, may contribute to thrombus formation and increase the risk of bleeding.

Endothelial cells play a crucial role in regulating vascular tone, preventing clot formation, and inhibiting inflammation. However, in patients with vascular disease, these functions are compromised. Endothelial dysfunction creates a pro-thrombotic state, increasing the likelihood of clotting and thereby elevating the risk of thrombosis. Additionally, this dysfunction diminishes the natural anticoagulant properties of the endothelium, further contributing to thrombus formation. Additionally, damage to the endothelium can weaken blood vessels, heightening the risk of bleeding [33,34,35].

Additionally, patients with a history of vascular disorders often have multiple comorbidities, and the medications used to manage these comorbidities may further increase the risk of bleeding [36]. Our study specifically focused on drugs flagged for concomitant use alerts in the package insert, such as ADPR inhibitors, aspirin, and P-glycoprotein inhibitors. However, recent studies on bleeding risk factors in Japanese patients using DOACs have highlighted the concomitant use of proton pump inhibitors, nonsteroidal anti-inflammatory drugs, and antiplatelet agents as potential risk factors for central nervous system or gastrointestinal bleeding [37]. Therefore, the presence of other concomitant medications may indeed influence the risk of bleeding in elderly patients.

Although the analysis of rivaroxaban revealed a significantly elevated HR for bleeding events in patients with a history of vascular disorders, there were no discernible differences in the background characteristics of these patients in the rivaroxaban-treated roup compared to those in other DOAC-treated groups. Of the 12 patients who developed bleeding events in the rivaroxaban group, none were treated with aspirin or ADP inhibitors. This may be attributed to the lack of detailed data collection on patient demographic characteristics and concomitant medication use in our study. Further accumulation and scrutiny of such information are necessary to better understand the impact of these factors.

This observational retrospective cohort study utilized medical information from medical records, and there are several limitations. First, the study included non-randomized data, and some patients may no longer attend the hospital in question, leading to incomplete follow-up and missing event data. Second, the study may have been terminated before capturing all relevant events. Third, the dataset may not include data, such as prescriptions from other hospitals. Fourth, the study’s results and discussion were based on data from 2016 to 2018 and did not include the most recent information. However, in Japan, the guidelines for NVAF treatment were revised in 2020 and are still in use. There were no significant changes in the drug treatment system for NVAF between the 2020 guidelines and prior ones, so we believe that the findings of this study will still contribute to future medical care.

Fifth, endpoint data, including stroke, systemic embolism, and bleeding, were not verified. The data were collected from a medical database using ICD codes and were not cross-referenced with individual medical records. Finally, important data on factors such as the type of AF (paroxysmal, persistent, or permanent), duration of illness, lifestyles (smoking and alcohol consumption), concomitant use of medications (beyond ADPR inhibitors, aspirin, and P-glycoprotein inhibitors), blood pressure, liver function, and the use of nonpharmacological therapies were not available in the dataset from the Mie-LIP-DB. These factors could act as confounding variables, and it is important to interpret the results of this study considering these limitations [38].

This study revealed that the cumulative incidence of bleeding events and SEEs with the use of DOACs followed a similar trend to that observed with warfarin. These results suggest that the overall efficacy and safety of DOAC treatment were generally comparable to that of warfarin in medical practice for NVAF patients over 75 in Japan. Additionally, the study identified an increased risk of bleeding in patients with a history of vascular disorders, including myocardial infarction, peripheral arterial disease, and aortic plaque. Although a history of vascular disorders is not specifically identified as a bleeding risk factor in the current package inserts or clinical guidelines, it is recommended to consider this medical history for patients aged 75 years or older.

## Figures and Tables

**Table 1:** Drug information for direct oral anticoagulant agents [6,7,8,9,10,17,18,19,20,24,25,26,27].

Generic Name		Edoxaban	Apixaban	Dabigatran Etexilate Methanesulfonate	Rivaroxaban
Classification		Factor Xa inhibitor		Direct thrombin inhibitor	Factor Xa inhibitor
Year of approval in Japan		2014	2013	2011	2012
Dosage and administration	Usual dose	60 mg once daily	5 mg twice daily	150 mg twice daily	15 mg once daily
	Reduced dosage and indications	30 mg once daily Body weight ≤ 60 kg Ccr ≤ 50 mL/min	2.5 mg twice daily Patients who meet at least two of the following criteria >80 years Body weight ≤ 60 kg Serum creatinine ≥ 1.5 mg/dL	110 mg twice daily History of gastrointestinal bleeding Ccr ≤ 50 mL/min	10 mg once daily Ccr ≤ 49 mL/min
	Elderly dose	15 mg once daily * Elderly patients at a high risk of bleeding	-	-	-
	Contraindications	Ccr ≤ 15 mL/min	Ccr ≤ 15 mL/min	Ccr ≤ 30 mL/min	Ccr ≤ 15 mL/min
Principal study for application approval		ENGAGE AF TIMI 48 [8]	ARISTOTLE [7]	RELY [5]	ROCKET-AF [6]
Patient’s characteristics in the DOAC part of the study	Age	72 years	70 years	72 years	73 years
	Body weight	<60 kg in approximately 10% of patients	Average 82 kg	Average 83 kg	BMI < 28.3
	Kidney function	Ccr ≤ 50 mL/min in 20% of patients	Ccr ≤ 50 mL/min in 16.5% of patients	Ccr ≤ 50 mL/min in about 29% of patients	Ccr ≤ 67 mL/min

* Edoxaban was approved in 2021 in 15 mg tablets, with an additional dosage option for older patients at higher risk of bleeding. BMI: body mass index; Ccr: creatine clearance.

**Table 2:** Patients’ characteristics.

Characteristics			Edoxaban	Apixaban	Dabigatran	Rivaroxaban	Warfarin
Number of cases			428	586	105	202	466
Follow-up period, days (interquartile range)			324.50 (253.75, 443.50)	360.00 (262.25, 526.50)	329.00 (250.00, 495.00)	372.00 (283.00, 564.00)	381.50 (276.00, 538.00)
Age, years			82 (78, 87)	83 (79, 87)	81 (77, 85)	81 (77, 86)	83 (79, 86)
Weight, kg			50.00 (43.18, 56.20)	49.70 (44.10, 57.10)	53.40 (47.20, 69.30)	51.80 (44.60, 59.45)	54.40 (44.55, 66.15)
Men			175 (40.9)	276 (47.1)	58 (55.2)	106 (52.5)	244 (52.4)
Ccr, mL/min			43.67 (34.88, 53.77)	40.83 (32.84, 54.03)	50.11 (39.76, 62.55)	42.55 (34.50, 52.92)	43.09 (29.55, 58.97)
Heart failure			115 (26.9)	165 (28.2)	18 (17.1)	45 (22.3)	153 (32.8)
Stroke/TIA			85 (19.9)	120 (20.5)	20 (19.0)	40 (19.8)	68 (14.6)
Diabetes mellitus			187 (43.7)	168 (28.7)	20 (19.0)	81 (40.1)	156 (33.5)
GI bleeding			4 (0.9)	5 (0.9)	0 (0.0)	2 (1.0)	7 (1.5)
Hypertension			274 (64.0)	391 (66.7)	60 (57.1)	119 (58.9)	313 (67.2)
Vascular disease			94 (22.0)	83 (14.2)	12 (11.4)	34 (16.8)	129 (27.7)
Dosage	Standard		46 (10.7)	194 (33.1)	3 (2.9)	84 (41.6)	-
	Reduced		382 (89.3)	392 (66.9)	102 (97.1)	118 (58.4)	-
ADPR inhibitor use			41 (9.6)	62 (10.6)	6 (5.7)	14 (6.9)	45 (9.7)
Aspirin use			50 (11.7)	81 (13.8)	9 (8.6)	24 (11.9)	79 (17.0)
P-glycoprotein inhibitor use			63 (14.7)	51 (8.7)	11 (10.5)	20 (9.9)	46 (9.9)
CHADS_2_ score (%)		1	177 (41.4)	292 (49.8)	64 (61.0)	87 (43.1)	205 (44.0)
		2	149 (34.8)	222 (37.9)	31 (29.5)	81 (40.1)	178 (38.2)
		3	83 (19.4)	59 (10.1)	9 (8.6)	24 (11.9)	61 (13.1)
		4	18 (4.2)	11 (1.9)	1 (1.0)	10 (5.0)	21 (4.5)
		5	0	2 (0.3)	0	0	1 (0.2)
		6	1 (0.2)	0	0	0	0

Values are presented as medians [interquartile ranges (Q1, Q3)] or *n* (%). ADPR, adenosine diphosphate receptor; Ccr, creatinine clearance; GI, gastrointestinal; TIA, transient ischemic attacks. CHADS_2_ scoring system assigning one point each for congestive heart failure, hypertension, age ≥ 75 and diabetes mellitus, and two points for prior stroke or transient ischemic attack.

**Table 3:** Incidence rate of bleeding events over 180 days.

	N	Number of Event Incidents at 180 Days	Event Yncidence Rate at 180 Days (%)	95% Confidence Interval
Warfarin	466	16	3.4	2.1–5.5
DOACs	1321	43	3.3	2.4–4.4
Edoxaban	428	14	3.3	2.0–5.5
Apixaban	586	20	3.4	2.2–5.2
Dabigatran	105	2	1.9	0.5–7.4
Rivaroxaban	202	7	4.0	2.0–7.8

N: Number of cases, DOACs: direct oral anticoagulants.

**Table 4:** Incidence rate of systemic embolism events over 180 days.

	N	Number of Event Incidents at 180 Days	Event Incidence Rate (%) at 180 Days	95% Confidence Interval
Warfarin	466	29	4.3	2.8–6.6
DOACs	1321	46	1.8	1.2–2.7
Edoxaban	428	17	2.3	1.3–4.3
Apixaban	586	22	2.0	1.2–3.6
Dabigatran	105	1	0.1	0.1–6.6
Rivaroxaban	202	6	0.5	0.1–3.5

N: Number of cases, DOACs: direct oral anticoagulants.

**Table 5:** Multivariate Cox regression analysis of patient characteristics for bleeding events *.

	Risk Factors	Hazard Ratio	95% Confidence Interval	*p*-Value
DOACs	Vascular disorders	1.83	1.16–2.88	<0.01
Edoxaban	Ccr levels	1.04	1.00–1.07	0.03
	Stroke/TIA	2.75	1.12–6.75	0.03
Rivaroxaban	Vascular disorders	5.18	1.67–16.09	<0.01

* Only factors with *p*-values less than 0.05 in the multivariate Cox regression analysis are shown. DOACs: direct oral anticoagulants, TIA: Transient Ischemic Attack.

## Data Availability

The usage regulations for the database used in this research do not permit the use of individual anonymized data or lists for purposes other than the submitted research plan. Therefore, the data from this research will not be used for secondary purposes or provided to other organizations.

## Supplementary Materials

The following supporting information can be downloaded at: https://www.mdpi.com/article/10.3390/pharmaceutics16121515/s1, Figure S1: Kaplan–Meier curves for the cumulative incidence up to 1000 days of bleeding events; Figure S2: Kaplan–Meier curves for the cumulative incidence up to 1000 days of systemic embolism events; Table S1: Definitions of systemic embolic events and bleeding events (These events were defined according to the previous studies [39,40]); Table S2: The types of bleeding observed in each treatment group; Table S3: The types of systemic embolism observed in each treatment group.
