# Supplementary material for: Systemic Embolism and Clinically Significant Bleeding Events in Older Adults with Nonvalvular Atrial Fibrillation After Treatment with Direct Oral Anticoagulants and Warfarin: A Retrospective Cohort Study in Japan
Source: Pharmaceutics. 2024 Nov 25;16(12):1515. doi: 10.3390/pharmaceutics16121515 (PMC11676222; doi:10.3390/pharmaceutics16121515)
Supplement: Supplementary file 1 [file pharmaceutics-16-01515-s001.zip › Table S1.pdf]

Table S1. Definitions of systemic embolic events and bleeding events

These events were defined according to the previous studies [1,2].

(1) Systemic embolic events

Definition of Systemic Embolism Events

ICD-10 code	ICD10 name
H34	Retinal vascular occlusion
I21.9	Acute myocardial infarction, unspecified
I24.0	Coronary thrombosis, not resulting in myocardial infarction
I74	Arterial embolism and thrombosis
K55.0	Acute vascular disorders of intestine
N28.0	Ischemia and infarction of kidney

Definition of retinal vascular occlusion (date of onset: date of ophthalmologic examination)

1. Ophthalmologic examination
   Ophthalmologic examination performed after the first day of follow-up (including the day of follow-up)
   Except for the second or subsequent ophthalmologic examination during the same hospitalization
2. Ophthalmologic examination and same month: diagnosis
   The patient was diagnosed with "H34: retinal vascular occlusion" in the same month as the ophthalmologic examination.

Definitions other than retinal vascular occlusion (date of onset: date of imaging)

1. Imaging Studies
   MRI or CT or X-ray (with contrast) or angio-echo after the first day of follow-up (including the day)
   Except for the second and subsequent imaging examinations during the same Hospitalization
2. Imaging and same month: Diagnosis
   Systemic embolism was diagnosed as a diagnosis during hospitalization (including the name of the hospitalization event or injury) on the same month as the imaging test date.
3. Imaging test day or the following day: Action
   ・Heparin 5,000 units or more or thrombectomy performed on the day of imaging or the following day (OR)
   ・Death within 30 days of the date of imaging
4. Calculation of emergency medical care management add-on or specific intensive care management add-on on the day of imaging examination

(2) Bleeding events

Definition of bleeding events

Bleeding events	Conditions with expression
Bleeding requiring transfusion	Transfusion of more than 800 mL
Intracranial hemorrhage	ICD codes I60-I63 (cerebrovascular diseases) and S06 (intracranial injuries)
Intraocular hemorrhage	ICD codes H00-H59 (eye and adnexal disorders) and S05.1 (globe and orbital tissue contusion
Upper gastrointestinal hemorrhage	ICD codes K20-K31 (esophageal, gastric, and duodenal diseases, excluding non-hemorrhagic or perforated conditions K25.3, K25.7, K25.9, K26.3, K26.7, K26.9, K27.7, K27.9, K28.7, K28.9) and K92 (other diseases of digestive system), I85.0 (esophageal varices with bleeding), I86.4 (gastric varices)
Lower gastrointestinal hemorrhage	ICD codes K55-K63 (other intestinal diseases), K65-K67 (peritoneal diseases), K92 (other diseases of digestive system), N98.8 (hemoperitoneum after egg collection)

Definition of bleeding requiring blood transfusion (date of onset: date of blood transfusion)

1. History of blood transfusion
   No history of blood transfusion within 30 days before the first day of follow-up (regardless of the amount of transfusion).
2. Amount of blood transfused
   Blood transfusion more than 800 mL after the first day of follow-up (including the day of follow-up)
3. Surgery
   No surgical intervention other than endoscopic hemostatic surgery was performed on the day before or on the day of the transfusion.
4. Excluding blood transfusions on the day of surgery or the following day.

Definition of intracranial hemorrhage (date of onset: date of imaging)

The following criteria have to be full-filled. However, the first criteria apply only when an event occurs during the hospitalization period after the starting day of follow-up (including the day of follow-up).

1. Medical history
   The following ICD10 names of injury and illness have not occurred after the first day of follow-up (including the day of follow-up):
   ・Cerebrovascular diseases (I60-63), but not sequelae (excluding I690, I691, I694)
   ・Intracranial injury (S06)

2. Imaging Studies

   MRI or CT scan performed after the first day of follow-up (including the day of follow-up), except for the second and subsequent imaging examinations during the same hospitalization

3. Name of injury or illness

   The following ICD10 names of injury and illness occurred on the same month as the date of imaging examination

   ・Cerebrovascular diseases (I60-63), but not sequelae (I690, I691, I694)

   ・Intracranial injury (S06)

4. Rehabilitation for cerebrovascular disease, etc.

   Meet any of the following

   ・Rehabilitation of cerebrovascular disease etc. within 30 days from the day of imaging examination

   ・Death within 30 days of imaging date

Definition of Intraocular Hemorrhage (Onset Day: Study Day)

1. Examination

   Ophthalmological examination performed after the first day of follow-up (including the day of follow-up)

   Except for the second or subsequent ophthalmologic examination during the same hospitalization

2. Name of injury or illness

   In the same month as the ophthalmological examination, the following ICD10 names of injuries and illnesses occurred

   ・Eye and adnexal disorders (H00-H59)

   ・Contusion of eyelid and peribulbar region (S00.1)

   ・Contusion of globe and orbital tissue (S05.1)

Definition of upper gastrointestinal bleeding (date of onset: date of examination)

1. Examination

   Gastroduodenal fiber or fecal occult blood test performed after the first day of follow-up (including the day of follow-up), except for the second and subsequent examinations during the same hospitalization

2. Name of injury or illness

   In the same month as the above examination, the following ICD10 names of injury and illness occurred

   ・Esophageal, gastric and duodenal diseases (K20-K31)

   However, those without hemorrhage or perforation (K25.3, K25.7, K25.9, K26.3, K26.7, K26.9, K27.7, K27.9, K28.7, K28.9) but not

・Other Diseases of the Digestive System (K92)
・Esophageal varices with bleeding (I85.0)
・Gastric varix (I86.4)
・Melena (K92.1)

3. Anticoagulants

No anticoagulant prescription on the day after testing

Definition of lower gastrointestinal bleeding (date of onset: date of examination)

1. Examination
   Fiberoptic colonoscopy or fecal occult blood test performed after the first day of follow-up (including the day), except for the second and subsequent examinations during the same hospitalization
2. Name of injury or illness
   In the same month as the above examination, the following ICD10 names of injury and illness occurred
   ・Miscellaneous Intestinal Diseases (K55-K63)
   ・Diseases of the Peritoneum (K65-K67)
   ・Other Diseases of the Digestive System (K92)
   ・Intraperitoneal hemorrhage after egg collection (N98.8)
3. Anticoagulants
   No anticoagulant prescription on the day after testing

References

1. Koretsune, Y.; Yamashita, T.; Yasaka, M.; Oda, E.; Matsubayashi, D.; Ota, K.; Kobayashi, M.; Matsushita, Y.; Kaburagi, J.; Ibusuki, K.; et al. Usefulness of a healthcare database for epidemiological research in atrial fibrillation. *J. Cardiol.* **2017**, *70*, 169–179.
2. Yamaguchi, T.; Fuji, T.; Akagi, M.; Abe, Y.; Nakamura, M.; Yamada, N.; Oda, E.; Matsubayashi, D.; Ota, K.; Kobayashi, M.; et al. The Epidemiological Study of Venous Thromboembolism and Bleeding Events Using a Japanese Healthcare Database—Validation Study. *Jpn. J. Drug Inform.* **2015**, *17*, 87–93.
