# Supplementary figures and images for: Systemic Embolism and Clinically Significant Bleeding Events in Older Adults with Nonvalvular Atrial Fibrillation After Treatment with Direct Oral Anticoagulants and Warfarin: A Retrospective Cohort Study in Japan
Source: Pharmaceutics. 2024 Nov 25;16(12):1515. doi: 10.3390/pharmaceutics16121515 (PMC11676222; doi:10.3390/pharmaceutics16121515)

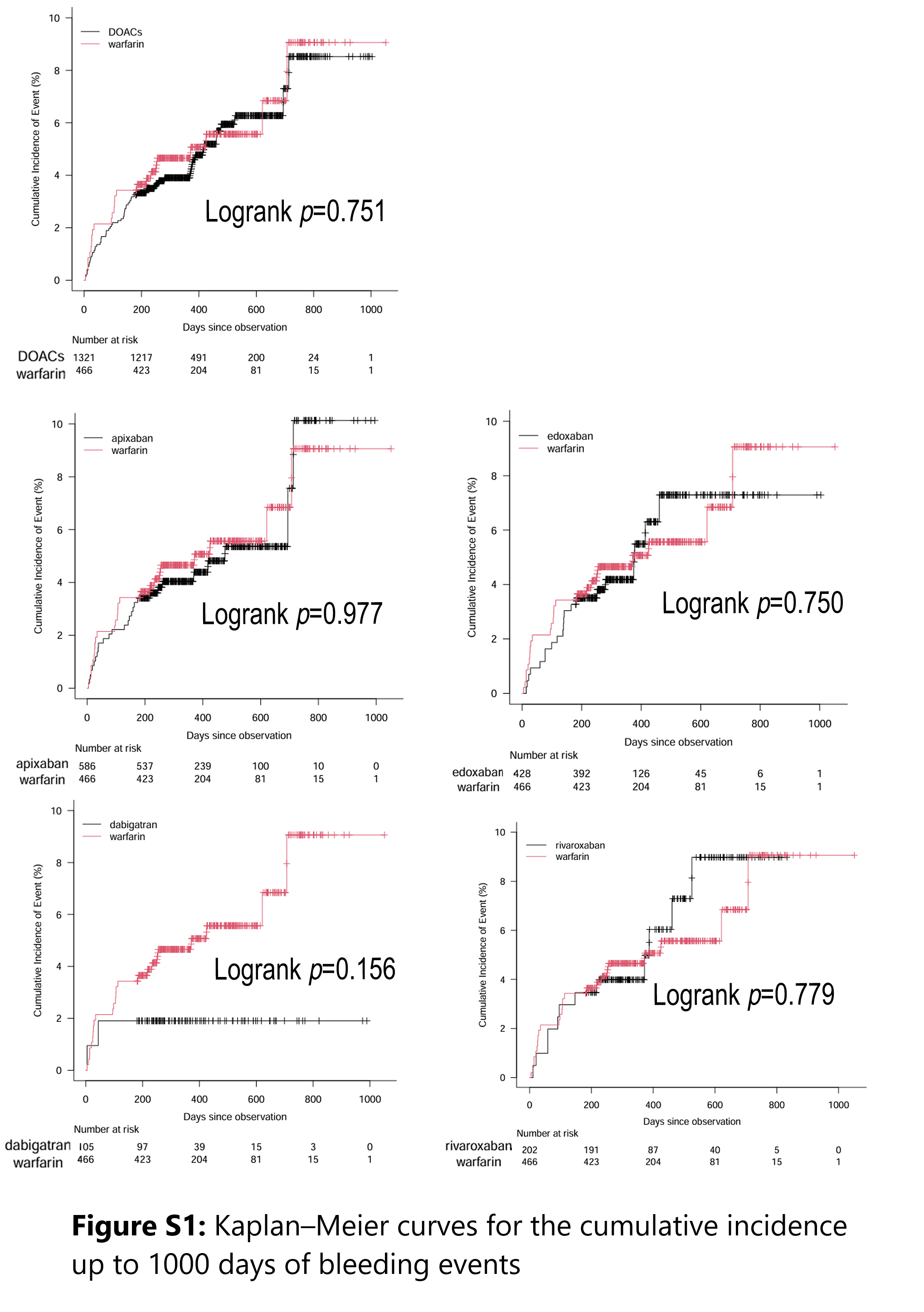

Supplement: Supplementary file 1 [file pharmaceutics-16-01515-s001.zip › Figure S1.tif]

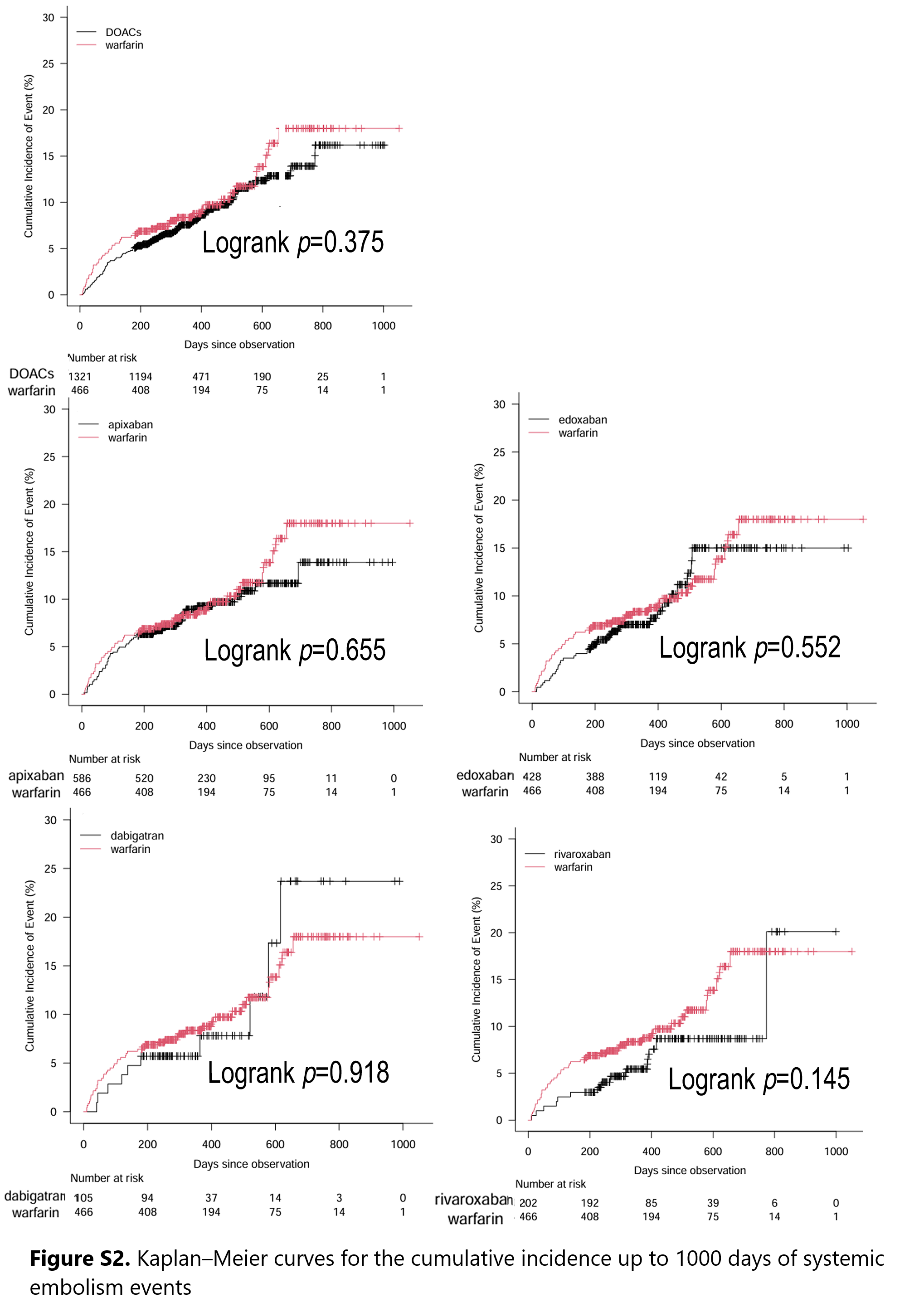

Supplement: Supplementary file 1 [file pharmaceutics-16-01515-s001.zip › Figure S2.tif]
